# Supplementary material for: Differential expression of the glucose transporter gene glcH in response to glucose and light in marine picocyanobacteria
Source: PeerJ. 2019 Jan 11;6:e6248. doi: 10.7717/peerj.6248 (PMC6330958; doi:10.7717/peerj.6248)
Supplement: Supplemental Information 1 [file peerj-07-6248-s001.docx]

*glcH* fragment from Prochlorococcus sp. strain_TAK9803-2.

5' TTACTGCATCCCATATCTTTATTAACATTAATAATGAACCTGCAATTATTACtgGtAAACCAGCAGAAATAAAGAATTTGAACAGAAAAAAACCAAATTGCGTCGCGACTAAACCTgtGCCTGCATCTCCAAGtCCATAAGAGAGCAT

3'
